# Safety and Efficacy of ^177^Lutetium-PSMA-617 Radioligand Therapy Shortly after Failing ^223^Radium-Dichloride

**DOI:** 10.3390/cancers14030557

**Published:** 2022-01-22

**Authors:** Justus Baumgarten, Daniel Groener, Christina Nguyen Ngoc, Nicolai Mader, Maximilian Chaurasia, Karen Davis, Jennifer Wichert, Felix K. H. Chun, Nikolaos Tselis, Christian Happel, Frank Grünwald, Amir Sabet

**Affiliations:** 1 Department of Nuclear Medicine, University Hospital Frankfurt, Theodor Stern Kai 7, 60590 Frankfurt, Germany; justus.baumgarten@kgu.de (J.B.); daniel.groener@kgu.de (D.G.); christina.nguyen@kgu.de (C.N.N.); nicolai.mader@kgu.de (N.M.); maxchaurasia@hotmail.com (M.C.); karen.davis@kgu.de (K.D.); Jennifer.wichert@kgu.de (J.W.); christian.happel@kgu.de (C.H.); frank.gruenwald@kgu.de (F.G.); 2 Department of Urology, University Hospital Frankfurt, Theodor Stern Kai 7, 60590 Frankfurt, Germany; felix.chun@kgu.de; 3 Department of Radiation Oncology, University Hospital Frankfurt, Theodor Stern Kai 7, 60590 Frankfurt, Germany; nikolaos.tselis@kgu.de

**Keywords:** PSMA, ^177^Lu-PSMA-617, ^223^Radium-dichloride, mCRPC

## Abstract

**Simple Summary:**

The alpha emitter ^223^Radium-dichloride (^223^Ra) and the beta emitter ^177^Lutetium (^177^Lu) targeting the prostate-specific membrane antigen (PSMA) are sequentially used for therapy of advanced bone-metastatic castration-resistant prostate cancer. Despite routine performance in patients who had received ^223^Ra, it is not clear whether disease sites refractory to alpha radiation can be effectively treated with ^177^Lu-PSMA-617. Secondly, it remains to be elucidated if therapy with ^177^Lu-PSMA-617 can be safely performed shortly after ^223^Ra, bearing in mind the myelotoxic potential of both treatments. The aim of our retrospective study was to evaluate safety and efficacy of ^177^Lu-PSMA-617 within less than 8 weeks after failing ^223^Ra. Radioligand therapy with ^177^Lu-PSMA-617 shortly after failing ^223^Ra is effective and can result in long-standing disease control even in patients with disseminated or diffuse bone involvement. Patients with oligo- and multifocal bone metastases show significantly longer overall survival with lower risk of significant hematotoxicity compared to patients with disseminated/diffuse involvement.

**Abstract:**

Bone-seeking ^223^Radium-dichloride (^223^Ra) is an established treatment prolonging survival and reducing morbidity in selected patients with metastatic castration-resistant prostate cancer (mCRPC) with skeletal involvement. Radioligand therapy with ^177^Lutetium-PSMA-617 (^177^Lu-PSMA-617) has been increasingly implemented in patients with mCRPC failing conventional treatment options. In this study, the safety and efficacy of ^177^Lu-PSMA-617 in patients with progressive bone involvement under treatment with ^223^Ra was assessed. Twenty-eight men (median age 73 years, range 63–89 years) with progressive mCRPC, who started ^177^Lu-PSMA-617 within 8 weeks after the last ^223^Ra administration, received a median of 4 (IQR 3–6) and a total of 120 cycles of ^223^Ra and a median of 4 (IQR 2–7) cycles ^177^Lu-PSMA-617 with a mean treatment activity of 6.5 ± 1.2 GBq per cycle, reaching a mean cumulative activity of 30.7 ± 23.4 GBq. A PSA response (≥50% PSA decline 12 weeks after the first ^177^Lu-PSMA-617 cycle) was observed in 18/28 (64.3%) patients and imaging-based partial remission (PR) was observed in 11/28 (39.3%) patients. Median imaging-based progression-free survival (PFS) was 10 (95% CI, 6–14) months and median overall survival (OS) was 18 (95% CI, 14–22) months. Patients with low bone tumor burden (2–20 lesions) had a significantly longer OS (28 vs. 14 months, *p* < 0.045) compared to patients with a high tumor burden (>20 lesions). Grade ≥ 3 hematological toxicity was observed in six patients after their last treatment cycle with anemia, leukopenia and thrombocytopenia in 5/28 (17.9%), 4/28 (14.3%) and 6/28 (21.4%) patients, respectively. In progressive bone-metastatic mCRPC patients, prompt initiation of ^177^Lu-PSMA-617 after failing ^223^Ra is effective with an acceptable toxicity profile.

## 1. Introduction

Many patients with advanced prostate cancer under androgen deprivation therapy (ADT) will eventually develop castration resistance and distant metastases, mostly to the bone [1,2]. Bone metastases may lead to complications including pain, fractures, hypercalcemia, spinal cord compression and impairment of bone marrow reserve resulting in higher morbidity and mortality [3]. Symptomatic relief may be achieved by analgesics, palliative radiotherapy and bisphosphonates/denosumab but only limited life prolonging therapeutic options exist, including androgen-receptor-signaling inhibitors (e.g., abiraterone, enzalutamide), taxane-based chemotherapy (docetaxel, cabazitaxel) and treatment with ^223^Radium dichloride (^223^Ra) [4,5,6,7].

Radionuclide therapy with the bone-seeking alpha emitter ^223^Ra is an established treatment prolonging survival and reducing morbidity in patients with osteoblastic bone dominant disease and no visceral metastases [8]. Radioligand therapy (RLT) targeting the prostate-specific membrane antigen (PSMA) has been increasingly implemented in patients with metastatic castration-resistant prostate cancer (mCRPC) failing standard treatment options [9,10]. PSMA is a type II transmembrane glycoprotein, highly overexpressed in prostate cancer cells. Using small-molecule inhibitors such as PSMA-617 labeled with the beta-emitting radionuclide ^177^Lutetium (^177^Lu) allows selective radiation of PSMA-expressing cells, whilst sparing surrounding normal tissue, regardless of metastatic site.

Several published studies on RLT with ^177^Lutetium-PSMA-617 (^177^Lu-PSMA-617) included patients previously treated with ^223^Ra and few studies reported the safety of ^177^Lu-PSMA-617 in such patients [11,12,13]. However, no study has addressed the feasibility of ^177^Lu-PSMA-617 directly after failure of ^223^Ra. It is not yet clear if bone metastases refractory to ^223^Ra can be effectively treated with ^177^Lu-PSMA-617. Furthermore, the safety of ^177^Lu-PSMA-617 immediately after ^223^Ra needs to be investigated, considering the myelotoxic potential of both treatments. This study aims to assess the safety and efficacy of ^177^Lu-PSMA-617 of patients with progressive bone involvement under treatment with ^223^Ra starting within 8 weeks after the last ^223^Ra application.

## 2. Materials and Methods

### 2.1. Patients

Twenty-eight men (median age 73 years, range 63–89 years) with progressive mCRPC, who started ^177^Lu-PSMA-617 within 8 weeks after the last ^223^Ra administration, were included in this retrospective study. Progression was assessed by bone scan routinely performed after the 3rd and the 6th cycle or in case of biochemical progression. A median of 4 (IQR 3–6) and a total of 120 cycles of ^223^Ra were administered with a mean activity of 4.197 ± 0.590 MBq, and no acute renal or bone marrow toxicities were observed. Other previous treatments comprised androgen receptor signaling inhibitors (abiraterone, enzalutamide) and taxane-based chemotherapy (Table 1).

Treatment initiation was approved by an interdisciplinary tumor board. General prerequisites for ^177^Lu-PSMA-617 included an estimated glomerular filtration rate (eGRF) of ≥30 mL/min/1.73 m^2^, hemoglobin (Hb) ≥ 8 g/dL, a white blood cell count (WBC) ≥ 2000/µL, platelets (Plt) ≥ 75,000/µL and sufficient PSMA-expression on ^68^Ga-PSMA-11 PET/CT imaging. The extent was categorized into (1) low (oligo-/multifocal, i.e., 2–20 lesions) and (2) high (disseminated/diffuse, i.e., >20 lesions). Written informed consent was given prior to each treatment cycle. Patient characteristics are depicted in Table 2.

### 2.2. ^177^Lutetium-PSMA-617 Radioligand Therapy

Inpatient treatments were carried out every 6–8 weeks using standard activities of 6–7.4 GBq ^177^Lu-PSMA-617. PSMA-617 ligand was supplied by ABX (Advanced Biochemical Compounds GmbH, Radeberg, Germany). Labeling was performed in-house with ^177^LuCl_3_ (ITM Isotopen Technologien München AG, Garching/Munich, Germany) [14].

### 2.3. Response Assessment

PSMA-based imaging was performed every 2–3 cycles for imaging based response assessment according to a recent consensus [15], as follows: progressive disease (PD: ≥2 new lesions or tumor volume/uptake >30%), stable disease (SD: tumor volume/uptake ± ≤30%; no new lesions), and partial response (PR: reduction in tumor volume/uptake >30%). Biochemical response assessment was based on changes in prostate-specific antigen (PSA) 12 weeks after treatment initiation, as follows: progression (≥25% increase exceeding 2 ng/mL, confirmed by a second measurement ≥3 weeks apart) according to criteria set out by the Prostate Cancer Working Group 3 (PCWG3) [16], response (≥50% decline 12 weeks after treatment initiation), and stable (values between <50% decline and <25% increase).

### 2.4. Toxicity Assessment

Blood values (Hb, WBC and Plt), PSA and renal function based on eGFR were tested prior to each cycle, every 4 weeks throughout the treatment and every 6–12 weeks after the last cycle. Common Terminology Criteria for Adverse Events (CTCAE), version 5.0 was used to grade hematologic adverse events. Grade ≥ 3 toxicities were denominated significant.

### 2.5. Data Analysis

Retrospective data analysis was approved by the local ethics committee and was carried out using SPSS (IBM SPSS Statistics 27.0, Armonk, NY, USA). The significance level of all tests was set at *p* < 0.05. Continuous variables are presented as median with interquartile range (IQR) or mean ± standard deviation. Categorical variables are shown as frequencies and percentages. Progression-free survival (PFS) is defined as the time interval from ^177^Lu-PSMA-617 initiation until progression on molecular, PSMA-based imaging, or the onset of other systemic treatments. Overall survival (OS) is defined as the time between the initiation of radioligand therapy and death from any cause; censoring was performed if the patient was alive at the time of analysis. The Kaplan–Meier method was used to calculate PFS and OS. Cox regression analysis was used to test the association of treatment response and survival.

## 3. Results

For 28 patients showing progressive bone-metastatic disease under ^223^Ra, radioligand therapy (RLT) with ^177^Lu-PSMA-617 was initiated within 5 ± 3 weeks after the last ^223^Ra administration. Three patients developed visceral metastases under ^223^Ra (one patient with adrenal, one with pulmonary and one with splenic metastases, respectively). Upon initiation of ^177^Lu-PSMA-617, 16 patients had low (oligo-/multifocal; ≤20 lesions) and 12 patients had high (disseminiated/diffuse; >20 lesions) bone tumor burden (based on PROMISE miTNM and previous studies) [17,18,19].

### 3.1. Response and Survival

A total of 131 treatment cycles were performed at intervals of 6 to 8 weeks. Patients received a median of four cycles (IQR 2–5) ^177^Lu-PSMA-617 with a mean activity of 6.5 ± 1.2 GBq per cycle, reaching a mean cumulative activity of 30.7 ± 23.4 GBq. A PSA decline of ≥50% 12 weeks after the first treatment cycle was seen in 18/28 (64.3%) patients, 7/28 (25.0%) patients showed a >25% PSA increase.

**Figure 1:**
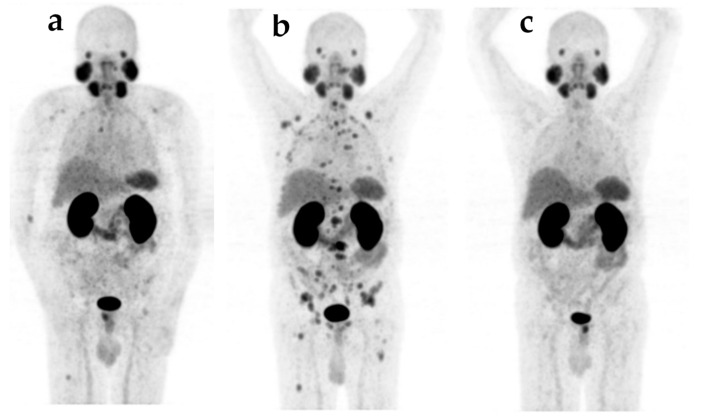
Maximum intensity projections of ^68^Ga-PSMA-11 PET/CT imaging in a 72-year-old patient (**a**) before ^223^Ra, (**b**) PD at baseline after ^223^Ra failure and (**c**) PR after 3 cycles of ^177^Lu-PSMA-617 and 25.3 GBq of cumulative treatment activity.

Imaging-based response to ^177^Lu-PSMA-617 consisted of PR in 11/28 (39.3%) patients, SD in 8/28 (28.6%) patients, and PD in 9/28 (32.1%) patients. An example of the disease course on ^68^Ga-PSMA-11 PET/CT imaging is provided in Figure 1.

**Figure 2:**
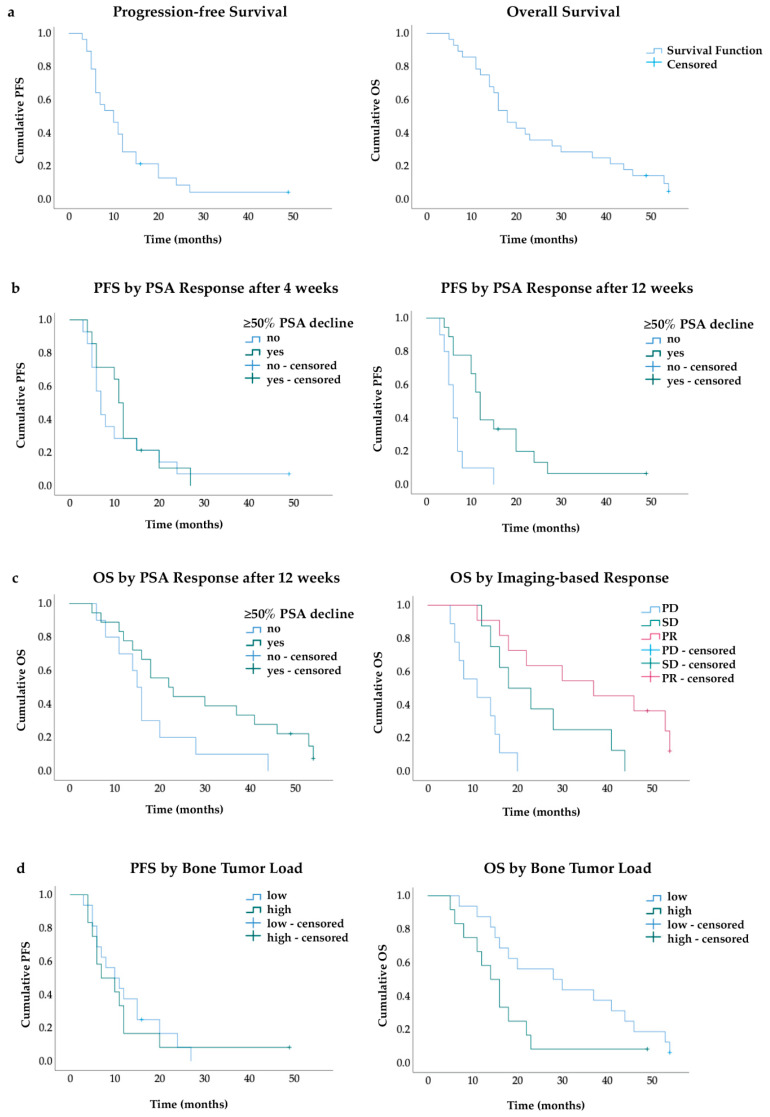
Kaplan–Meier curves (**a**) OS and PFS in whole cohort (**b**) PFS by PSA response after 4 and 12 weeks (**c**) OS by PSA and imaging-based response (**d**) PFS and OS by bone tumor load; PFS: progression-free survival, OS: overall survival, PR: partial response, SD: stable disease, PD: progressive disease.

The median PFS for the whole study cohort was 10 (95% CI 6–14) months and the OS was 18 (95% CI 14–22) months (Figure 2a). PSA-responders (≥50% PSA decline after 12 weeks) showed a significantly longer PFS than non-responders (12 vs. 6 months; *p* = 0.001) as well as longer OS (22 vs. 15 months; *p* = 0.047) (Figure 2c). In contrast, early ≥50% PSA decline after 4 weeks, observed in 14/28 (50%) patients, was not a predictor for longer survival, as follows: PFS (11 vs. 7 months; *p* = 0.589) and OS (16 vs. 18 months; *p* = 0.894) (Figure 2b).

Two patients were alive after a mean follow-up period of 25 ± 16 months. Patients with objective imaging-based PR showed a significantly longer OS of 37 (95% CI 11–63) months than patients with SD (95% CI 8–28) 18 months and PD 11 (95% CI 2–20; *p* < 0.001) months (Figure 2c). Disease control (SD + PR) also resulted in significantly longer OS (28 vs. 11 months, *p* < 0.001) and PFS (12 vs. 5 months, *p* < 0.001). Patients with disseminated/diffuse bone involvement at baseline (*n* = 12) showed a significantly shorter OS (28 vs. 14 months, *p* = 0.045), despite similar PFS (10 vs. 7 months; *p* = 0.701) compared to patients with oligo-/multifocal bone metastases (*n* = 16) (Figure 2d).

In total, 17 out of 28 (60.7%) patients received subsequent therapies upon progression (Table 1), 13 of whom after initial disease control (7 PR, 6 SD). They lived significantly longer than those who did not receive further life-prolonging treatment (28 vs. 12 months; *p* = 0.026).

### 3.2. Safety

At baseline, 24/28 (85.6%) patients had low grade anemia, 7/28 (25.0%) leukopenia and 4/28 (14.3%) thrombocytopenia (Table 3).

Hb, WBC, and Plt showed a minor, yet significant absolute decline through the course of ^177^Lu-PSMA-617. Mean Hb changed from 12.1 ± 1.4 to 10.1 ± 2.1 g/dL (*p* = 0.001), WBC from 5.2 ± 1.9 to 4.0 ± 1.6 × 10^9^/L (*p* < 0.001), and Plt from 189 ± 51 to 125 ± 74 × 10^9^/L (*p* = 0.011) (Table 4).

Overall, 6 out of 28 (21.4%) patients developed significant hematotoxicity (grade ≥ 3) after their last ^177^Lu-PSMA-617 cycle with anemia in 5 (17.9%), leukopenia in 4 (14.3%) and thrombocytopenia in 6 (21.4%) necessitating transfusion therapies. Overall, 5 out of 6 had high bone tumor burden at baseline.

All six patients with significant hematotoxicity also showed disease progression which resulted in treatment discontinuation. One out of six patients received abiraterone and re-treatment with enzalutamide and lived 11 months after the last cycle. Another patient received docetaxel and repeated transfusion therapy and lived 13 months. All other patients died within 4 months after the last treatment cycle. Characteristics of these patients are detailed in Table 5.

At baseline, 15/28 (53.6%) patients showed low grade renal function impairment (grade 1/2). Moderate function decline to grade 2 was seen in 7/28 (25.0%) patients but no significant nephrotoxicity occurred.

## 4. Discussion

The rationale of systemic treatments with the bone-seeking calcium mimetic ^223^Radium-dichloride and PSMA-targeting radioligand ^177^Lutetium-PSMA-617 is the selective internal radiation of prostate cancer cells. Despite being a common scenario in clinical practice, only few studies focused on the outcome of ^177^Lu-PSMA-617 in patients previously treated with ^223^Ra and no report exists on the efficacy of RLT with ^177^Lu-PSMA-617 in patients refractory to ^223^Ra. The observed PFS of 10 months and OS of 18 months in this retrospective study demonstrate efficacy of RLT with ^177^Lu-PSMA-617 in mCRPC patients failing ^223^Ra with progressive bone metastases.

Following several retrospective studies on the value of ^177^Lu-PSMA-617 in patients with mCRPC [14,20], the recently published phase III VISION study showed a significant life-prolonging effect of ^177^Lu-PSMA-617 when added to standard care in patients with progressive mCRPC after exhausting at least one taxane-based chemotherapy regimen and one androgen-receptor-pathway inhibitor [9]. Patients treated with ^177^Lu-PSMA-617 and standard care had a longer PFS (8.7 vs. 3.4 months, *p* < 0.001) and OS (15.3 vs. 11.3 months, *p* < 0.001) as compared to standard care alone. The influence of resistance to ^223^Ra was not evaluated as patients receiving ^223^Ra within 6 months of randomization were excluded.

Few reports suggested that survival outcome of patients undergoing ^177^Lu-PSMA-617 may not be affected by previous exposure to ^223^Ra but it is unclear if ^177^Lu-PSMA-617 could produce any objective response in patients failing ^223^Ra. In a recent study by Sartor et al., 26 patients receiving 1 to 6 (median 6) ^223^Ra administrations 1 to 31 months (median 8 months) before the start of ^177^Lu-PSMA-617 were analyzed [11]. Thirteen patients received other life prolonging treatment between ^223^Ra and ^177^Lu-PSMA-617. An OS of 13.2 (95% CI 8.4–16.2) months was reported but no information on response to ^177^Lu-PSMA-617 and PFS was given. In another multicenter study (WARMTH), ^223^Ra 1–36.4 months before the first cycle of ^177^Lu-PSMA-617 had no impact on OS (10.8 vs. 11.3 months, *p* = 0.34) [21]. The median time interval of 3.9 months between the two treatments may be indicative of resistance to ^223^Ra in some patients, but no information was given regarding the outcome of ^177^Lu-PSMA-617 in patients failing ^223^Ra. No further information was provided regarding the outcome of ^177^Lu-PSMA in this subgroup. In our study, RLT with ^177^Lu-PSMA-617 starting within 8 weeks from ^223^Ra could yield long-lasting disease control (PR + SD) in 19/28 (67.9%) patients with a PFS of 12 (95% CI 11–13) months. This finding indicates the efficacy of ^177^Lu-PSMA-617 in patients with progressive bone-metastatic mCRPC irrespective of recent resistance to ^223^Ra. Disease control was also associated with a significantly longer OS (28 vs. 11 months, *p* < 0.001). The relatively long OS even in non-responders may be due to subsequent treatments after ^177^Lu-PSMA-617 in most patients (*n* = 5/9) with a significant life-prolonging effect.

PSA response, defined as ≥50% PSA reduction at 12 weeks, has also been used in other studies for treatment response assessment in patients receiving ^177^Lu-PSMA-617 [22,23]. In our study, PSA response showed significantly longer survival (PFS 12 vs. 6 months, *p* = 0.001; OS22 vs. 15 months, *p* = 0.047). The PSA response rate of 64.3% in our cohort is in line with results from the phase II TheraP trial showing ≥50% PSA decline after ^177^Lu-PSMA-617 in 66% (65/99) of mCRPC patients previously treated with docetaxel; no information regarding previous treatment with ^223^Ra and PFS in PSA responders was reported [24]. In a study by Ahmadzadehfar et al., PSA response was not significantly different between patients with no history of treatment with ^223^Ra (*n* = 17/26; 58.6%) compared to patients treated with 1 to 6 cycles ^223^Ra within up to one year (median: 11 weeks) before the start of ^177^Lu-PSMA-617 (*n* = 9/20; 45.0%). The significance of initial PSA response (≥50% after 4 weeks) to ^177^Lu-PSMA-617 as a positive predictor of outcome remains controversial. A few studies in heterogeneous patient cohorts showed a longer OS in patients with early PSA response [13,21,25], whereas other studies reported no significantly longer survival in early responders [23,26]. In a study by Leibowitz et al. on 24 elderly patients at >75 years of age, 14 of whom previously treated with ^223^Ra, a PSA decline of ≥ 50% was associated with a significantly longer OS (10.9 vs. 3.1 months, *p =* 0.0006) [13]. In our study, patients (*n* = 14/28, 50.0%) with PSA response after the first cycle showed only a trend towards longer imaging-based PFS (11 vs. 7 months; *p* = 0.589).

^223^Ra has a relatively low myelosuppressive potential, possibly owing to the short range (<100 μm) of high-energy alpha particles and its quick blood extraction [27]. In the pivotal ALSYMPCA trial, grade ≥ 3 reduction in blood parameters was observed in 13% (Hb), 6% (Plt), and 3% (WBC) of patients. Treatment with *^177^*Lu-PSMA-617 has a comparably low toxicity profile due to direct binding to the cell membrane and internalization, sparing the surrounding tissue [9]. Radiation exposure of ^223^Ra to the kidneys is extremely low, as the major route of elimination is the bowel. ^177^Lu-PSMA-617 has demonstrated low nephrotoxicity due to its rapid renal excretion. In our patients, no grade ≥ 3 nephrotoxicity occurred.

Previous studies reported grade ≥ 3 hematologic adverse events in the range of 8–24% for anemia, 0–8% for leukopenia and 1–11% for thrombocytopenia [20,24,28,29]. In the VISION study, 68/529 (12.9%) patients developed grade ≥ 3 anemia, 42 (7.9%) thrombocytopenia and 13 (2.5%) leukopenia [9]. In a recent study by Groener et al., significant hematologic adverse events were observed in a total of 13/140 patients (9.3%) after ^177^Lu-PSMA-617 [19]. Here, previous treatment with ^223^Ra was not significantly associated with occurrence of hematotoxicity (OR: 0.93, 95% CI 0.27–3.20, *p* = 0.58). In our collective of patients receiving ^177^Lu-PSMA-617 immediately after ^223^Ra, significant hematotoxicity occurred more frequently (21.4%). However, this may be only partially explained by the short time interval between the two treatments. In a preliminary study, Sartor et al. observed similarly high rate of hematotoxicity under ^177^Lu-PSMA-617 in patients treated with ^223^Ra a median of 8 months before starting ^177^Lu-PSMA-617 [11]. Here, 5/26 patients (19.2%) showed significant hematotoxicity throughout ^177^Lu-PSMA-617, especially grade 3 anemia. The authors suggested increasing tumor load as an underlying factor.

High extent of metastatic bone involvement may increase the risk of myelotoxicity and is suggested as a negative prognostic factor for survival after RLT with ^177^Lu-PSMA-617. Groener et al. showed, that patients with disseminated/diffuse osseous involvement at baseline (*n* = 77/140) had a significantly higher incidence of hematotoxicity (OR 5.08, *p* = 0.04) [19]. Similarly, in a report on the subgroup of patients from the multicenter WARMTH study with known numbers of bone lesions, only patients with >20 bone lesions or diffuse bone marrow involvement developed significant thrombocytopenia (*n* = 18), no significant leukopenia was observed [30]. In our study, five out of six patients developing significant hematotoxicity had >20 bone lesions at baseline. Patients presenting with high bone tumor load (*n* = 12/28) also showed a significantly shorter OS compared to patients with low bone tumor load (*p* = 0.045) despite similar PFS (*p* = 0.701).

Our study has clear limitations. Firstly, similar to previous studies, using standard activities for all patients may be considered as a confounding factor in the evaluation of response to treatment and survival outcome. The retrospective nature of our study and the small cohort inevitably limit the strength of the results and impede extensive subgroup analysis. Moreover, patients with temporary myelotoxicity of grade ≥ 3 who recovered later than 8 weeks after the last ^223^Ra treatment were not included in this analysis. Subsequent therapies contributed to longer OS; therefore, PFS may be a more appropriate surrogate for evaluation of treatment efficacy in our study.

## 5. Conclusions

RLT with ^177^Lu-PSMA-617 can be effectively and safely initiated as early as 8 weeks after failure of ^223^Ra in patients with progressive bone-metastatic mCRPC refractory to ^223^Ra. Objective response can be achieved even in patients with more advanced disease and disseminated/diffuse bone metastases, albeit with increasing incidence of significant hematotoxicity.

## Figures and Tables

**Table 1:** Systemic mCRPC treatments in 28 patients.

Treatment	Prior to ^223^Ra	After ^177^Lu-PSMA-617
Abiraterone	16 (57)	4 (14)
Enzalutamide	10 (36)	5 (18)
Docetaxel	9 (32)	8 (29)
Cabazitaxel	0 (0)	1 (3)
Re-treatment ^177^Lu-PSMA-617	-	7 (25)

**Table 2:** Patient characteristics.

Variable	Before ^223^Ra	Before RLT
PSA (ng/mL)	35.2 (15.9–147)	161 (76–336)
Hemoglobin (g/L)	13.6 (12.6–14.3)	12.1 (11.2–13.3)
White blood cells (10^9^/L)	5.5 (4.6–7.4)	5.2 (4.0–7.0)
Platelets (10^9^/L)	192 (224–276)	189 (169–240)
Sites of metastases		
Bone		
-oligofocal/multifocal	18 (64)	16 (57)
-disseminiated/diffuse	10 (36)	12 (43)
Local recurrence	3 (11)	8 (29)
Lymph nodes	16 (57)	20 (71)
Visceral	0 (0)	3 (11)

Data presented as median with interquartile range (IQR) or *n* (%), PSA: prostate-specific antigen.

**Table 3:** Baseline and intra-/post-therapeutic hematologic toxicity grades based on CTCAE v5.0.

Toxicity	Prior to ^223^Ra (Grade)				Prior to RLT (Grade)				Post RLT (Grade)			
	1	2	3	4	1	2	3	4	1	2	3	4
Anemia	13 (46)	1 (4)	0 (0)	0 (0)	23 (82)	1 (3)	0 (0)	0 (0)	15 (54)	8 (29)	5 (18)	0 (0)
Leukopenia	1 (4)	0 (0)	0 (0)	0 (0)	5 (18)	2 (7)	0 (0)	0 (0)	6 (21)	4 (18)	3 (11)	1 (4)
Thrombocytopenia	0 (0)	1 (4)	0 (0)	0 (0)	4 (14)	0 (0)	0 (0)	0 (0)	12 (43)	0 (0)	2 (7)	4 (14)

Data are *n* (%).

**Table 4:** Median hemoglobin (Hb), white blood cell counts (WBC) and platelets (Plt) with standard deviation prior to ^223^Radium-dichloride (^223^Ra), at baseline, upon maximum deterioration and in follow-up.

Blood Parameter	Prior to ^223^Ra	Prior to RLT	Lowest Post RLT	Follow-Up
Hb (g/L)	13.6 ± 1.4	12.1 ± 1.4	10.1 ± 2.1	10.8 ± 2.1
WBC (10^9^/L)	5.5 ± 1.8	5.2 ± 1.9	4.0 ± 1.6	4.6 ± 1.9
Plt (10^9^/L)	224 ± 64	189 ± 51	125 ± 74	128 ± 88

**Table 5:** Previous therapies and course of six patients with grade ≥ 3 hematologic adverse events.

Patient	Previous Therapies after Castration Resistance	Toxicity (CTC_max_)			Course of Treatment/Disease after RLT
		Hb	WBC	Plt	
1	ENZA, ABI, ^223^Ra	3	4	4	transfusion (RBC, BP), PD, death 15 weeks after last cycle
2	DOCE, ABI, ^223^Ra	3	4	4	transfusion (RBC, BP), PD, death 18 weeks after last cycle
3	ABI, ^223^Ra	3	3	4	transfusion (RBC, BP), PD, death 15 weeks after last cycle
4	^223^Ra	3	3	4	transfusion (RBC, BP), PD, DOCE, death 60 weeks after last cycle
5	DOCE, ABI, ENZA, ^223^Ra	2	3	3	transfusion (RBC), PD, death 14 weeks after last cycle
6	ENZA, ^223^Ra	3	3	2	transfusion (RBC, BP), PD, Re-ENZA, ABI, death 48 weeks after last cycle

DOCE: docetaxel, ABI: abiraterone, ENZA: enzalutamide, RBC: packed red blood cells, BP: blood platelet concentrates, Hb: hemoglobin, WBC: white blood cells, Plt: platelets.

## Data Availability

The datasets analyzed and/or analyzed during the current study are available from the corresponding author on reasonable request.
